# Exploring the Association between Retinal Nerve Fiber Layer Thickness and Initial Magnetic Resonance Imaging Findings in Patients with Acute Optic Neuritis

**DOI:** 10.1155/2011/289785

**Published:** 2011-05-29

**Authors:** Fiona Costello, William Hodge, Y. Irene Pan

**Affiliations:** ^1^Departments of Clinical Neurosciences and Surgery, Hotchkiss Brain Institute, University of Calgary, Calgary, AB, Canada T2N 2T9; ^2^Department of Ophthalmology, The University of Western Ontario, London, ON, Canada N6A 4V2; ^3^McGill University, Montreal, QC, Canada H3a

## Abstract

*Background*. Recent studies have shown that OCT-measured retinal nerve fiber layer (RNFL) values may represent a marker for axonal damage in the anterior visual pathway of optic neuritis (ON) and multiple sclerosis (MS) patients. The goal of this study was to determine the link between RNFL values and initial magnetic resonance imaging (MRI) evidence of central nervous system (CNS) inflammation in patients with acute ON. *Methods*. Fifty patients who experienced ON as a clinically isolated syndrome (CIS) were followed for a mean period of 34 months with OCT testing. RNFL values in affected (ON) eyes and clinically unaffected (non-ON) eyes were compared between patients with MRI evidence of white matter lesions and those with normal baseline MRI findings, over a two year period. *Findings*. Twenty-one patients (42%) developed clinically definite MS (CDMS) during the study. After two years, temporal RNFL values were thinner (*P* = .07) in ON patients with MRI lesions at baseline, but the results were not significant. *Conclusions*. There is no association between RNFL values and baseline MRI status in ON patients at risk for future CDMS over a two year period.

## 1. Introduction

Optic neuritis (ON) is an inflammatory optic nerve injury, which is strongly associated with multiple sclerosis (MS) [1–11]. Approximately 20% of MS patients present with ON as their first demyelinating event, and 30 to 70% of MS patients develop ON during the course of their disease [2, 7]. The longitudinal followup from the optic neuritis treatment trial [3] (ONTT) demonstrated that the initial magnetic resonance imaging (MRI) study is the most potent predictor for future MS after acute ON: such that evidence of disseminated central nervous system (CNS) inflammation on baseline MRI increases the risk for future clinically definite MS (CDMS) after acute ON [4–6]. After 15 years 72% of ON patients with one or more white matter lesions on their initial MRI developed CDMS, as compared to 25% of patients with no MRI lesions [6]. The administration of high-dose corticosteroids has been shown to delay the conversion to MS for up to two years after ON, but not thereafter [8]. Early initiation of disease-modifying therapies delays the development of MS in patients with ON and other clinically isolated syndromes (CISs) [9–11]. 

The evidence supporting early initiation of disease-modifying therapy in patients at risk for MS is robust [9–11], as is the need to develop effective, cost-effective strategies to capture disease activity and monitor therapeutic effects in these patients. One tool which has emerged as a potential structural marker for axonal loss in MS patients is optical coherence tomography (OCT) [7, 12–25]. This noninvasive ocular imaging technology employs low-coherence interferometry to generate high-resolution [<10 microns (*μ*m)], cross-sectional images of the retinal nerve fiber layer (RNFL) by measuring the backscatter of infrared light [26, 27]. The RNFL contains the retinal ganglion cell axons that comprise the optic nerve; and because it lacks myelin, represents a unique region of the central nervous system. Changes in RNFL thickness after ON have been interpreted to reflect initial axoplasmic flow stasis and subsequent attrition caused by inflammation in the anterior visual pathway. Recent studies have shown that OCT-measured RNFL values are reduced after ON [12–16, 25] and that the extent of RNFL atrophy correlates with diminished scores of visual and neurological function [12–16, 19, 21, 24, 25] MRI measures of optic nerve and brain atrophy [17–20], and disease activity in MS patients [19]. To date, an association between RNFL thickness and initial MRI status in ON patients at risk of developing MS has not been established. Thus, the primary objective of this study was to determine whether RNFL values differed between ON patients with evidence of clinically silent brain lesions on initial MRI and patients with no MRI evidence of CNS inflammation over a two-year period. Our second aim was to compare RNFL values between patients treated with high-dose corticosteroid therapy and untreated patients, to ascertain whether RNFL differences distinguished the two groups.

## 2. Methods

### 2.1. Study Design and Sampling

This was a prospective cohort study of consecutively sampled patients referred to the Ottawa Hospital Neuro-Ophthalmology Clinic for the evaluation of acute ON between January 2003 and June 2007. The study received approval by the local ethics committee at this institution, and participating patients provided informed written consent.

### 2.2. Inclusion and Exclusion Criteria

Fifty CIS patients who experienced a single, unilateral ON event were included in the study. Patients were diagnosed with ON if they demonstrated the following clinical features: decreased visual acuity, a visual field defect, which followed the topography of the RNFL, a relative afferent pupil defect, and a compatible fundus examination (mild or no optic disc edema and the absence of pallor at the time of the acute event). Exclusion criteria included other established causes of vision loss in the affected eye (amblyopia, glaucoma, and dense cataracts), a known diagnosis of MS or neuromyelitis optica (NMO), and inability to undergo reliable OCT testing.

**Other Variables**

Demographic and clinical variables including age, gender, the presence of pain, mono-focal (ON without other neurological symptoms) versus multi-focal ON (associated with neurological symptoms referable to a region of the CNS different from the anterior visual pathway) as a CIS presentation, and the initiation of disease-modifying disease (DMD) therapy were recorded. The time to baseline MRI acquisition and MRI protocols varied among patients. For this reason, we were not able to employ the revised McDonald criteria [28] to define radiological conversion to MS after ON Instead, we documented whether patients had MRI evidence of clinically silent lesions representing CNS inflammation at the time of the ON event [9]. All MRI results were interpreted by qualified neuroradiologists at the University of Ottawa. Specific MS MRI protocols were employed (1.5T GE scanners), which included (coronal, axial, and sagittal imaging) T1-weighted, T2-weighted, and FLAIR sequences, either with or without gadolinium. The treating physician employed individualized discretion regarding the decision to administer corticosteroid therapy for acute management of ON. Patients who received corticosteroid therapy were treated within two weeks of the ON event with the equivalent of 1000 mg intravenous methylprednisolone daily for three days. Disease-modifying therapies were administered to a minority (12/50) of patients during the course of this study. No patients initiated disease-modifying therapy earlier than 6 months after ON. The limited number of CIS patients who received disease-modifying agents precluded efforts to compare the effects of disease-modifying therapy on RNFL values after ON.

### 2.3. Clinical Assessment

Patients were followed with repeat visual and OCT testing for a minimum of 24 months. The neuroophthalmic assessment included best-corrected Snellen visual acuity, visual field analysis, and dilated ophthalmoscopy. Neurological evaluations were performed at 6-month intervals by a neurologist at the MS Clinic at the Ottawa Hospital. Patients aged greater than 45 years or those with atypical features (including incomplete recovery after ON) also underwent visual electrodiagnostic testing (including pattern visual evoked potential (VEP) and multi-focal electroretinogram studies) to exclude retinal mimics of ON. If clinically indicated, additional studies were also performed to exclude conditions that could mimic MS such as myeloproliferative disorders, NMO, syphilis, sarcoidosis, or Lyme disease.

### 2.4. Optical Coherence Tomography

The OCT (Stratus version 3; OCT 4 Software, Zeiss Meditech, Dublin, Calif, USA) system was used to obtain circular peripapillary scans (Fast RNFL protocol), which included a set of three 3.4 mm diameter retinal scans averaged to provide the RNFL thickness at 256 points along the circumference of the circular scan in each eye after mydriasis with 1% tropicamide. The OCT software employed an automated computerized algorithm to calculate the average thickness of the RNFL and to compare these measurements to a normative database of age-matched controls, for patients aged 18 years or older. A trained ophthalmic medical technologist performed all OCT testing and monitored scans to ensure that fixation was reliable. OCT scans with signal strength equal to or greater than 7 (out of 10) were included in the study.

### 2.5. Outcome Measures and Statistical Analysis

The primary outcome measures in this study were RNFL values in ON eyes and non-ON eyes, which were compared between patients with clinically silent lesions on their baseline MRI scan and patients with no baseline evidence of CNS inflammation. As a secondary outcome measure, we compared RNFL values between patients treated with high-dose corticosteroid therapy for acute ON and untreated patients. Continuous variables were first checked for normality, and then summary statistics were calculated and reported. Median and range were computed for continuous variables that demonstrated a non-Gaussian distribution, including counts and percentages for categorical variables. For group comparisons, either the Student's *t*-test or the Kruskal-Wallis rank sum test was used depending on the variable's distribution attribute. The entire statistical analysis was performed using STATA (v. 9, College Station, Tex, USA). 

## 3. Results

### 3.1. Demographics and Clinical Presentation

Fifty patients (100 eyes) were included in the study. The mean age was 34 years, and the mean follow-up period was 34 months (range 24–44 months). All patients were followed for a minimum of 24 months. Twenty-one patients (42%) developed CDMS during the course of the study, with a mean conversion time of 27 months. The baseline demographics and clinical data are included in Table 1.

### 3.2. Comparing Baseline MRI Status, Treatment with High-Dose Corticosteroids, and RNFL Thickness

*ON Eyes: * The presence or absence of disseminated white matter lesions on the baseline cranial MRI scan in patients was not associated with significant differences in RNFL thickness a year after ON. By the second year of followup, temporal RNFL values (49.8 *μ*m) were thinner (*P* = .07) in patients with abnormal baseline MRI scans, but the results did not statistically significant (Table 2). RNFL values were not significantly differ between patients treated with high-dose corticosteroid therapy and untreated patients for up to two years after acute ON (Table 2). *Non-ON Eyes *RNFL values did not differ between patients with clinically silent lesions on initial MRI and patients without initial MRI evidence of CNS inflammation for up to two years (Table 3). Similarly, treatment with high-dose corticosteroid therapy, or the lack thereof, was not associated with any significant differences in RNFL thickness for non-ON eyes at year one or year two of followup (Table 3). 

## 4. Discussion

In the current study, we observed no association between the presence of clinically silent lesions on baseline MRI and RNFL thickness in CIS patients followed for two years after an acute ON event. By the second year of followup, temporal RNFL values were thinner in ON-eyes of patients with abnormal baseline MRI scans, yet differences did not reach statistical significance (*P* = .07). One explanation for this observation is that there is discordance between OCT-measured axonal damage and MRI evidence of disseminated CNS inflammation in CIS patients. Conventional MRI protocols may reflect inflammatory activity in lieu of axonal pathology, particularly at the earliest stage of MS. Given that more robust correlations have been noted between RNFL atrophy in the anterior visual pathway and MRI measures of brain volume and atrophy [17, 19, 20], diffusion tensor imaging [29, 30], and magnetic transfer ratios [18], there may be a stronger correlation between OCT outcomes and evolving MRI protocols that are specifically aimed at capturing axonal attrition in the CNS. 

There were no differences in RNFL atrophy noted between patients treated with high-dose corticosteroids for acute ON and untreated patients in this study. It is noteworthy that we did not randomize ON patients into treatment protocols. It is therefore possible that there was some bias on the part of the treating physician that influenced the decision to administer or withhold treatment, which may have affected our results, Yet, the administration of high-dose corticosteroids does not impact visual recovery after ON or future risk of MS in the long term [8]; therefore, another potential explanation for our findings is that RNFL atrophy after acute ON is not significantly impacted by acute management with corticosteroid therapy.

Only two prior reports have explored whether OCT-measured RNFL values distinguish patients at future risk of CDMS after ON, and in both studies the results were predominantly negative [31, 32] Previously, we compared RNFL values in ON eyes and non-ON eyes between patients who developed CDMS (42%) and those that did not develop MS within a minimum of 24 months after an acute ON event (58%), in the same patient cohort. [31] Mean RNFL values were reduced in ON eyes of non-MS patients as compared to CDMS ON eyes after one year (*P* = .05) due to more severe ON events in the former [31]. Temporal RNFL values were lower in the non-ON eyes of CDMS patients, but the results were not statistically significant (*P* = .13) [31]. From our findings, we concluded that RNFL thickness did not differentiate patients at higher risk of converting to CDMS after ON [31]. Similarly, Outteryck and colleagues [32] recently performed OCT testing on 56 CIS patients (18 with optic neuritis and 38 without optic neuritis) and 32 control subjects, to investigate whether RNFL thickness and macular volume revealed early retinal axonal loss. In this prospective case series there was no link between RNFL atrophy and dissemination of CNS inflammatory lesions on the initial MRI, gadolinium enhancement on baseline MRI, multifocal presentation, abnormal visual evoked potentials, or the development of Mc-Donald proven MS at 6-months [32]. Furthermore-patients who developed CDMS (*n* = 13) or McDonald criteria-proven MS (*n* = 23) did not have more severe RNFL atrophy [32]. These investigators concluded that OCT does not demonstrate retinal atrophy at the earliest clinical stage of MS, nor does it predict conversion to MS at 6 months in CIS patients [32].

There were a number of shortcomings in our study, which may have impacted our results. First and foremost, our patient population was quite limited, which likely hindered the statistical power of our comparisons. Second, we used clinical criteria [33] and not radiological criteria [28] to confirm the diagnosis of MS, which may have diminished our sensitivity to detect the diagnosis of MS in some CIS patients. Third, we did not randomize ON patients into treatment protocols, and it is therefore possible that patients who received corticosteroids were treated due to some bias on the part of the treating physician. Fourth, the two-year followup period may have been insufficient to detect significant RNFL differences between ON patients with disseminated CNS inflammation on initial MRI and those without clinically silent MRI results at baseline. Therefore, further study is needed to determine the predictive value of RNFL thickness in ON patients at risk of future MS and to establish the role of OCT in the earliest phases of this disease. Future clinical trials should include larger patient cohorts and potentially employ novel MRI strategies, which are sensitive to the early detection of axonal attrition in MS. 

## Figures and Tables

**Table 1:** Demographic and clinical characteristics among optic neuritis patients.

Demographics/characteristics	
Number of patients	50
Mean age in years (range)	34 (18–50)
Male: female^‡^	10 : 40
CIS*: CDMS^†^	29 : 21
Pain (%)	42/50 (84)
Mono-focal^§^: Multi-focal (%)	18 : 32
Abnormal MRI^#^ (%)	32/50 (64)
Abnormal CSF** (%)	14/20 (70)
Corticosteroids^*∧*^ (%)	25/50 (50)

^‡^Ratio of male to female patients included in the study; ratio of * clinically isolated syndrome patients to ^†^clinically definite MS patients; number of patients presenting with ^§^mono-focal versus multi-focal optic neuritis as a CIS at baseline; ^10#^number and percentage of abnormal magnetic resonance imaging scans at presentation; ^9∗∗^ number and percentage of patients with positive oligoclonal bands in their cerebrospinal fluid analysis at baseline:  $\hat{\;}$  number and percentage of patients who received treatment with the equivalent of 1000 mg intravenous methylprednisolone for three days for acute optic neuritis.

**Table 2:** Comparing MRI status, corticosteroid therapy, and RNFL* values in optic-neuritis-affected eyes.

RNFL ON Eyes*	Year 1		*P* value	Year 2		*P*-value
	MRI+^§^ (*n* = 32)	MRI−^||^ (*n* = 18)		MRI+ (*n* = 32)	MRI− (*n* = 18)	
Overall (SD)^¶^	84.4 (17.3)	80.1 (21.1)	.45	80.9 (16.6)	85.8 (21.6)	.36
Superior	110.8 (22.3)	102.9 (31.9)	.32	106.4 (21.6)	106.6 (29.6)	.98
Inferior	106.3 (25.0)	95.8 (28.7)	.20	99.3 (29.2)	107.9 (33.0)	.35
Nasal	68.1 (19.8)	67.1 (21.7)	.87	67.3 (16.5)	70.1 (21.0)	.61
Temporal	52.4 (17.5)	53.9 (15.2)	.78	49.8 (14.2)	58.5 (18.7)	.07

	Steroid^#^ (*n* = 25)	Untreated** (*n* = 25)		Steroid (*n* = 25)	Untreated (*n* = 25)	

Overall (SD)	81.4 (15.8)	84.3 (21.4)	.59	81.8 (18.6)	83.4 (18.7)	.77
Superior	104.7 (22.2)	111.4 (29.7)	.38	104.5 (23.5)	108.5 (25.7)	.57
Inferior	100.7 (22.2)	104.5 (30.5)	.63	102.1 (31.3)	102.8 (30.5)	.94
Nasal	70.5 (21.7)	65.2 (18.6)	.37	67.8 (18.9)	68.8 (17.6)	.85
Temporal	49.5 (14.9)	56.2 (17.7)	.16	52.3 (15.4)	53.3 (17.5)	.88

*Retinal nerve fiber layer thickness (*μ*m); ^§^baseline magnetic resonance imaging evidence of clinically silent inflammation [8]; ^||^normal baseline magnetic resonance imaging scan; ^¶^mean overall RNFL values (standard deviation) and mean RNFL values in the superior, inferior, nasal, and temporal quadrants; ^#^patients treated with the equivalent of 1000 mg of intravenous methylprednisolone for acute management of optic neuritis; **patients not treated acutely for optic neuritis.

**Table 3:** Comparing MRI status, corticosteroid therapy, and RNFL* values in nonaffected eyes.

RNFL Non-ON Eyes*	Year 1		*P* value	Year 2		*P* value
	MRI+^§^ (*n* = 32)	MRI−^||^ (*n* = 18)		MRI+ (*n* = 32)	MRI− (*n* = 18)	
Overall (SD)^¶^	103.9 (10.5)	102.2 (9.4)	.56	104.1 (12.2)	104.3 (9.9)	.94
Superior	131.1 (15.6)	132.9 (13.0)	.67	134.4 (20.6)	135.3 (13.6)	.87
Inferior	134.0 (16.9)	125.4 (12.7)	.07	131.0 (17.9)	127 (14.9)	.42
Nasal	84.3 (17.0)	80.4 (17.7)	.46	84.0 (18.0)	84.7 (21.4)	.89
Temporal	66.3 (13.0)	70.0 (10.1)	.30	66.9 (13.6)	70.7 (13.8)	.34

	Steroid^#^ (*n* = 25)	Untreated** (*n* = 25)		Steroid (*n* = 25)	Untreated (*n* = 25)	

Overall (SD)	103.6 (9.3)	103.0 (11.0)	.84	104.7 (10.5)	103.6 (12.5)	.75
Superior	130.7 (12.4)	132.8 (16.7)	.63	136.0 (14.7)	133.5 (21.4)	.63
Inferior	131.4 (14.2)	130.5 (17.8)	.85	130.2 (17.8)	129.0 (16.2)	.80
Nasal	85.8 (18.6)	80.0 (15.5)	.23	82.8 (19.8)	85.7 (18.6)	.60
Temporal	66.6 (11.2)	68.6 (13.0)	.56	69.7 (13.8)	66.8 (13.7)	.47

*Retinal nerve fiber layer thickness (*μ*m); ^§^Abnormal baseline magnetic resonance imaging study [8]; ^||^Normal baseline magnetic resonance imaging study; ^¶^Mean overall RNFL value (standard deviation), and mean RNFL values in the superior, inferior, nasal and temporal quadrants; ^#^Patients treated with the equivalent of 1000 mg of intravenous methyl-prednisolone for acute management of optic neuritis; **Patients not treated acutely for optic neuritis.
